# Development of a Transient Magnetic Field Sensor Based on Digital Integration and Frequency Equalization

**DOI:** 10.3390/s21134268

**Published:** 2021-06-22

**Authors:** Hongzhi Ouyang, Xueling Yao, Jingliang Chen

**Affiliations:** 1 State Key Laboratory of Electrical Insulation and Power Equipment, Xi’an Jiaotong University, Xi’an 710049, China; oyhz2000@stu.xjtu.edn.cn (H.O.); cjl@mail.xjtu.edu.cn (J.C.); 2 School of Electrical Engineering, University of South China, Hengyang 421001, China

**Keywords:** magnetic field sensor, digital integration, frequency equalization, lightning electromagnetic environment, calibration

## Abstract

Transient magnetic field sensors are used in various electromagnetic environment measurement scenarios. In this paper, a novel magnetic field sensor based on a digital integrator was developed. The antenna was a small B-DOT loop. It was designed optimally for the simulation. The magnetic field signal was digitally integrated with the improved Al-Alaoui algorithm, resulting in less integration error. To compensate for the bandwidth loss of the optical fiber system, we specially designed an FIR (finite impulse response) filter for frequency compensation. The circuit was described, and the transimpedance amplifier was specially designed to ensure the low noise characteristic of the receiver. The sensitivity of the sensor was calibrated at 68.2 A·m^−1^/mV, the dynamic range was 50 dB (1–300 kA/m), the linear correlation coefficient was 0.96, and the bandwidth was greater than 100 MHz. It was tested and verified under the action of an A-type lightning current. The sensor exhibited high-precision performance and flat amplitude-frequency characteristics. Therefore, it is suitable for lightning positioning, partial discharge testing, electromagnetic compatibility management, and other applications.

## 1. Introduction

Electromagnetic pulse measurement is an important tool in lightning positioning, electromagnetic compatibility, and partial discharge research. The strong transient electromagnetic field environment caused by lightning strikes can interfere with or damage aircraft electromechanical systems—this is called the indirect lightning effect. Compared with traditional aluminum alloy and other metal materials, the composite material has poor electrical conductivity, which leads to a decrease in the electromagnetic shielding performance of the aircraft’s skin, which may make aircraft vulnerable to damage in extreme weather such as lightning and may cause catastrophic accidents. For the study of the electric and magnetic field distribution of key positions inside aircraft under a lightning electromagnetic environment, various types of electromagnetic field sensors have been developed worldwide in recent years. These sensors use antennas to induce electromagnetic pulse signals and electro-optical conversion technology to transmit measurement signals through optical fibers. This measurement method has a strong anti-interference ability and wide response frequency [1,2]. We are confident that the measurement range of electromagnetic sensors will be larger, the frequency band will be wider, and the sensitivity will be higher with the development of science and technology [3].

The magnetic field sensors have matured day by day. Baum proposed a variety of electromagnetic field sensors [4], and Kanda performed detailed calculations on them [5]. For magnetic field sensors, integration is generally required. Current magnetic field sensors use analog integrators or perform digital integration on an external computer. Analog integrators are generally composed of passive components or operational amplifiers. The integration error resides in the low- and high-frequency bands, and it is difficult to overcome integration drift [6]. Chao used the Mobius strip loop as the B-DOT antenna, then used the upper computer software for integration. The sensor’s bandwidth was expected at 500 MHz [7]. Kong also used the upper computer software for integration, using a multi-gap loop as B-DOT antenna, and the sampling rate of the sensor was able to reach up to 2 GS/s [8]. The integration on the external computer is not real-time and flexible, and it cannot solve the problem of DC drift, which is unfavorable for electromagnetic environment measurement. Jin used digital integration technology on the Rogowski coil so that the amplitude-frequency response error did not exceed 10^−3^ dB, and the phase-frequency error did not exceed 1° [9], whereas Han used a data acquisition card for digital processing and integration, and its error was low to 10^−4^% [10]. At present, digital integrators are used on low-frequency equipment such as Rogowski coils and current transformers. However, there are few reports on ns-level transient current or magnetic field measurement applications. There are still many issues worthy of study in terms of measurement accuracy and anti-interference capability.

Optical fiber systems can exhibit channel unevenness and low-pass characteristics, and equalization techniques are often used to offset part of the nonlinear fading in these systems. These techniques can be divided into hardware equalization technology and software equalization (digital equalization) technology. Hardware equalization technology is generally realized by a circuit using RC electronic components. Software equalization technology generally uses digital filters to equalize the signal path. Analog equalization has the disadvantages of limited bandwidth, weak anti-interference ability, and lack of flexibility and is not suitable for the transmission of transient signals. Wei designed a digital amplitude-frequency equalizer to make the power amplifier fluctuate less than 1 dB in the 20–20 kHz [11], whereas Kavehrad [12] and Falconer [13] used digital equalization for OFDM to improve communication efficiency. In indoor visible light communication, Long used carrier-free amplitude-phase modulation with digital filtering and frequency equalization technology to reduce the complexity in the system [14]. Digital equalization technology has been widely used in the communication field with its unique flexibility.

In response to the need for transient magnetic field measurements generated by lightning, we designed a B-DOT sensor with a rise time of fewer than 1 ns. It was combined with a digital integrator and optical fiber transmission system to construct a transient magnetic field measurement system with high precision. Carbon fiber-reinforced plastics (CFRP) and aluminum (AL) alloy materials were tested and verified. Section 2 introduces the working process of the system. Section 3 optimizes the design of the antenna. Section 4 introduces the digital integrator algorithm. Section 5 analyzes and designs the key circuits. Section 6 and Section 7 include calibrating and testing the sensor. Finally, a conclusion is provided in Section 8.

## 2. System Specification

**Figure 1:**
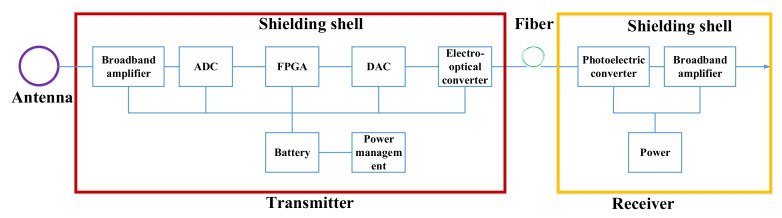
System block diagram.

The purpose of the system design is mainly to measure the electromagnetic radiation generated by lightning. As shown in Figure 1, the system is mainly composed of three parts: an antenna, a transmitter, and a receiver. After the magnetic field signal is received by the antenna, it undergoes broadband amplification, digital integration, and electro-optical conversion and is transmitted by a single-mode optical fiber to the receiver. The receiver contains a photoelectric conversion circuit and a broadband amplifying circuit, which finally sends the signal to an oscilloscope or spectrum analyzer for observation. To prevent electromagnetic interference, both the transmitter and receiver are shielded with thick aluminum alloy to ensure that the shielding effectiveness (SE) is greater than 100 dB. The energy of artificial lightning waves is mainly concentrated below a few hundred kHz, which is consistent with the energy spectrum distribution of natural lightning waves. To make the sensor applicable to a wider range, we set the bandwidth target at 100 MHz.

## 3. Modeling and Simulation of Antennas

**Figure 2:**
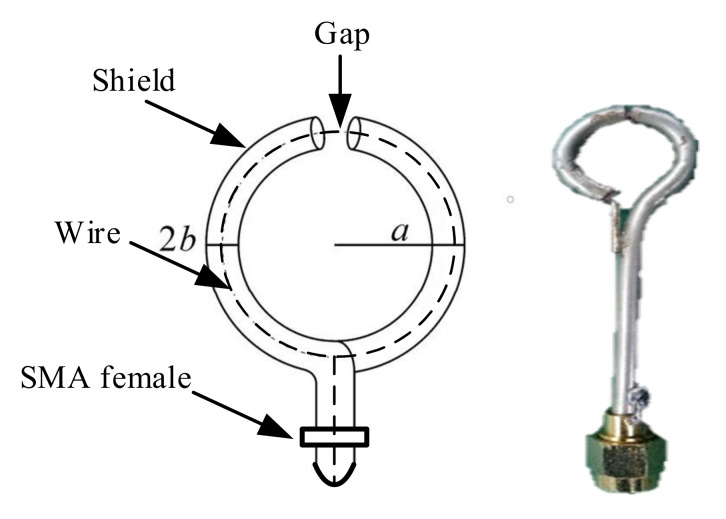
Structure and photo of the B-DOT.

Since a load of a magnetic field sensor is typically 50 Ω or 75 Ω (as determined by the characteristic impedance of the transmission cable), the turning frequency of the sensor is relatively high. Most magnetic field sensors measure the rate of change in a magnetic field, similar to a B-DOT sensor. As shown in Figure 2, to avoid differential output, we used a shielding ring sensor with unbalanced output. The induced voltage generated at the gap of the shielding ring leads to the load on the right half of the coaxial cable, and the output can be directly connected to the coaxial cable through an SMA connector. The radius of the ring is a, and b is the radius of the coaxial cable. The entire ring is a shielded symmetrical structure with strong anti-interference ability.

**Figure 3:**
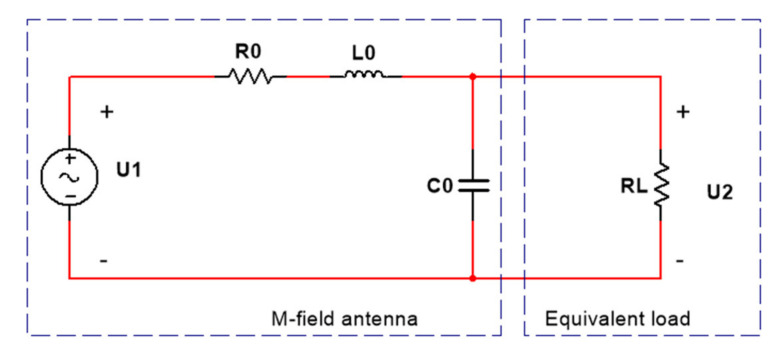
The equivalent circuit diagram of B-DOT.

The equivalent circuit of the antenna is shown in Figure 3 [15]. *U*_1_ is the voltage induced by the antenna, which is proportional to the equivalent magnetic flux; *R*_0_, *L*_0_, and *C*_0_ are the resistance, inductance, and capacitance of the antenna itself, respectively; *R*_L_ is the load resistance; and *U*_2_ is the voltage on the load terminal.

The transfer function is

(1) $$H(s)=\frac{U_{2}(s)}{U_{1}(s)}=\frac{1}{L_{0}C_{0}s^{2}+(R_{0}C_{0}+L_{0}/R_{L})s+(1+R_{0}/R_{L})}$$

where *s* is a complex variable.

By ignoring *R*_0_ and *C*_0_, there is

(2) $$H(s)=\frac{1}{\frac{L_{0}}{R_{L}}s+1}$$

The turning frequency is

(3) $$f_{0}=\frac{R_{L}}{2\pi L_{0}}$$

According to the frequency response characteristics, the turning frequency determines the measurement method of the impulse magnetic field. The B-DOT sensor can work in three modes: differential mode, transition mode, and self-integration mode. Only when the frequency of the measured magnetic field is lower than the turning frequency is the output signal of the antenna proportional to the differential of the magnetic field.

**Figure 4:**
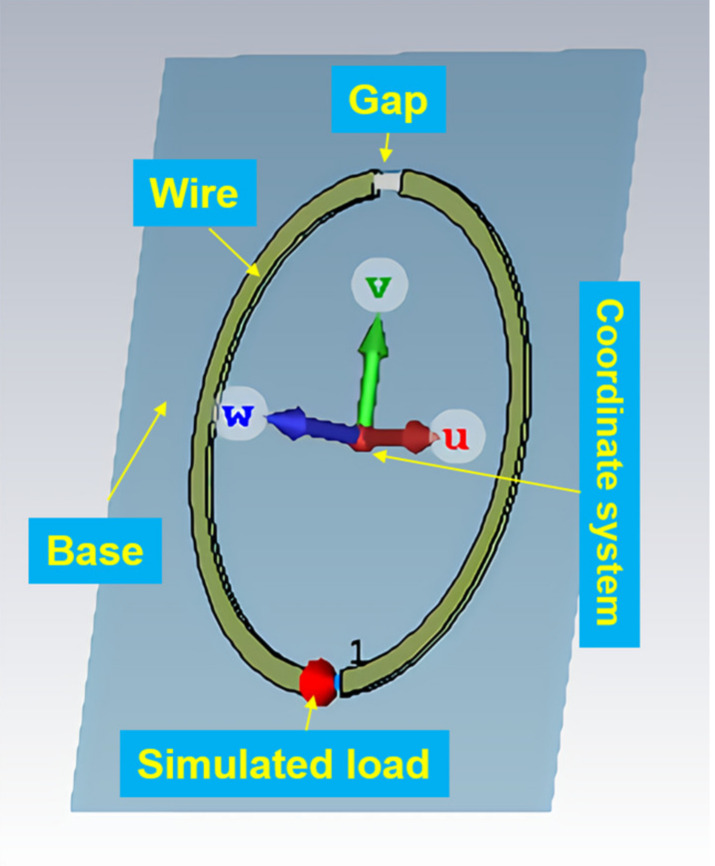
The model of antenna.

The antenna was simulated in the CST STUDIO 2017 microwave studio (Darmstadt, Germany). We mainly investigated the influence of antenna size and load on antenna performance [16]. As shown in Figure 4, the antenna was modeled first. The inner diameter of the loop antenna was 20 mm, and the outer diameter was 22 mm. The material included coaxial cable and oxygen-free copper. The base was rectangular and made of FR-4 epoxy glass fiber.

**Figure 5:**
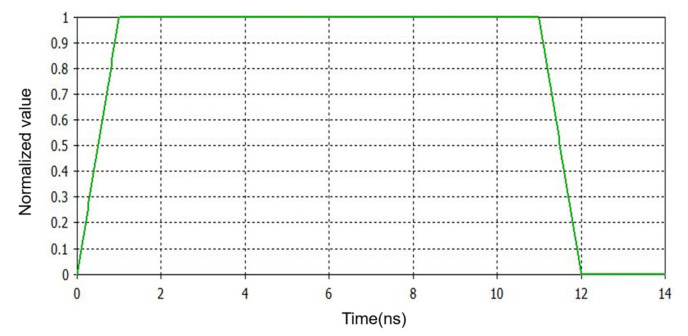
The plane wave.

We set a plane wave source with linear polarization. It was 0.3 m away in the w direction. A rectangular pulse wave was selected as the plane excitation wave; as shown in Figure 5, the rise time and fall time were both 1 ns, and the duration was 10 ns. The time range was set to 14 ns, and the frequency range was set to 0–100 MHz.

**Figure 6:**
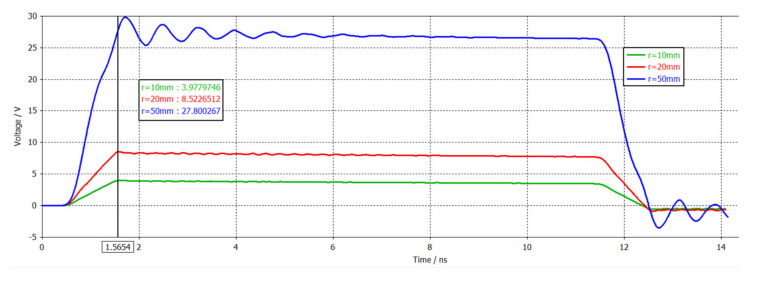
The relationship between the antenna size and the rise time (r = a).

It can be obtained by simulation: the upper limit frequency of the sensor was mainly determined by the size of the sensor. The larger the antenna diameter, the lower the upper limit frequency (corresponding to the rise time), as shown in Figure 6. The lower limit frequency of the sensor was mainly determined by the load resistance *R*_L_. The larger the values of *R*_L_, the lower the lower limit frequency. A photo of the antenna is shown in Figure 2 (a = 20 mm, b = 2 mm).

## 4. Improvement of Digital Integrator

**Figure 7:**
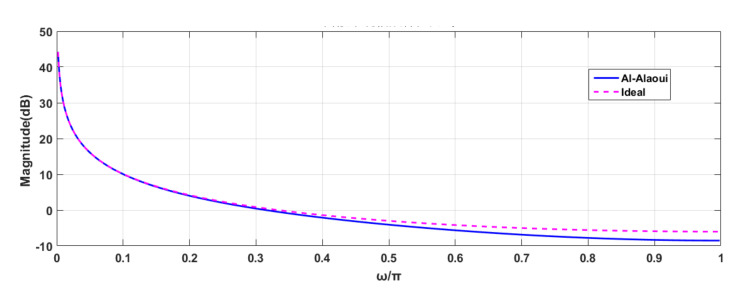
Amplitude-frequency curve with the comparison of improved Al-Alaoui integration algorithm and ideal integration algorithm.

The analog integrator relies too much on temperature drift and time drift characteristics and exhibits poor accuracy in low- and high-frequency bands. In contrast, digital integrators are stable, reliable, and highly repeatable. Common digital integration algorithms include rectangular, trapezoidal, and Simpson. Al-Alaoui integrated the trapezoidal and rectangular integral algorithms according to the ratio of 1:3 to obtain the Al-Alaoui integral algorithm. The transfer function is Equation (4), and *T* is the sampling interval [9,17]. The frequency characteristic curve of the Al-Alaoui algorithm is close to the ideal integral curve, but the error increases as the frequency increases, as shown in Figure 7.

(4) $$H(z)=\frac{5T}{8}•\frac{7z^{-2}+8z^{-7}+z^{-12}}{1-z^{-10}}$$

To solve the problem of DC drift in the input signal, we introduced the attenuation factor *k* value in the Al-Alaoui algorithm. The change in the *k* value will affect the integration error and adjustment time.

(5) $$H(z)=\frac{5T}{8}•\frac{7z^{-2}+8z^{-7}+z^{-12}}{1-kz^{-10}}$$

**Figure 8:**
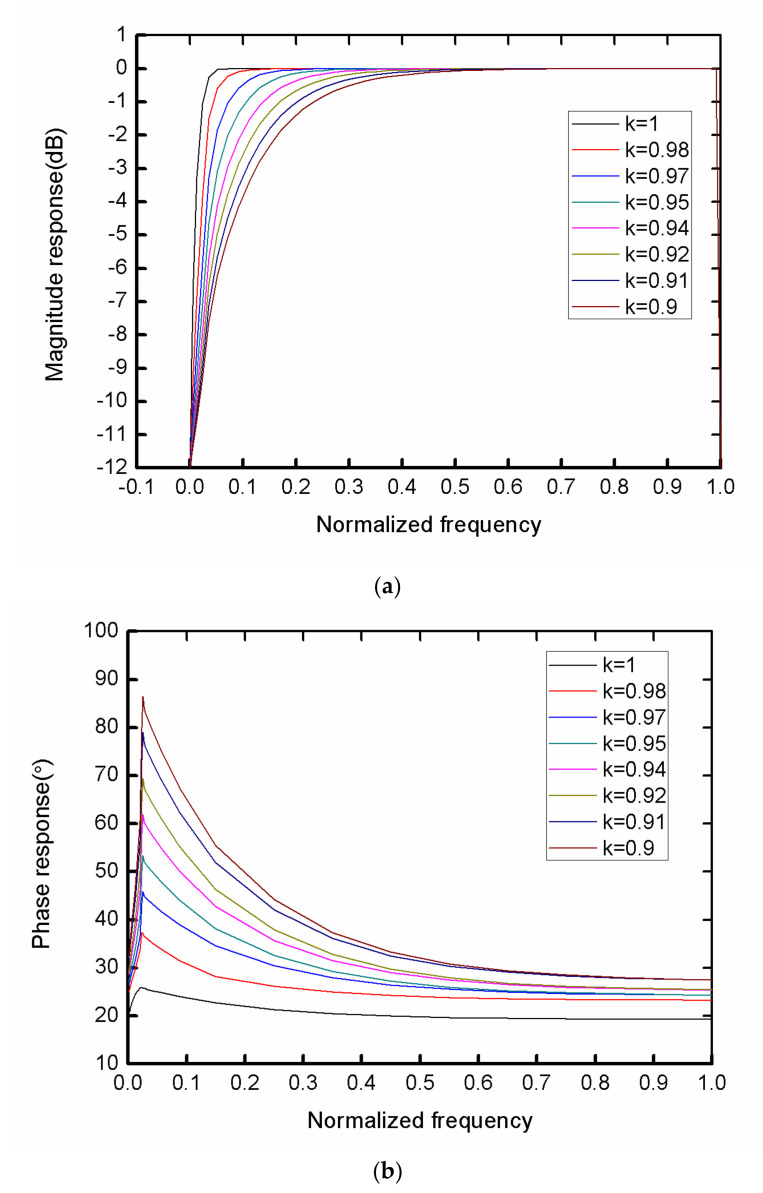
Frequency response corresponding to different *k* values with (**a**) amplitude-frequency response and (**b**) phase frequency-response.

To accurately illustrate the influence of *k* on the amplitude response error, we used MATLAB (MathWorks, 2015b, Natick, MA, United States) to calculate the response with different *k* values, and the result is shown in Figure 8. The larger the value of *k*, the smaller the error between the algorithm and the ideal algorithm, but the longer it will take to reach the steady state. At the same time, to a certain extent, the DC component caused by the initial value not being zero will be significantly attenuated effectively. Through the phase-frequency response curve, when *k* was from 0.98 to 1 and not 1, the high-frequency band was almost unaffected. Comprehensively, the *k* value was determined to be 0.98, and the amplitude-frequency error was less than 0.1 dB at this time.

**Figure 9:**
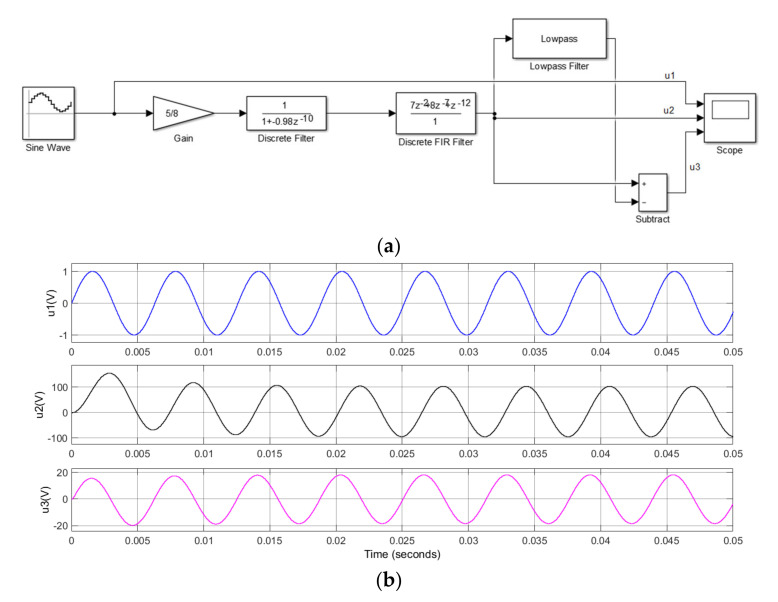
Comparison of integration effects with (**a**) Simulink model and (**b**) waveforms.

A time-domain circuit model was built to observe the effect of the integration algorithm when *k* = 0.98, as shown in Figure 9. Lowpass filter was used to simulate the frequency characteristics of the optical fiber system; T is 10 μs in Equation (5). In the module, u1 is a discrete sine wave, u2 is the signal after integration, and u3 is the output of the system. From the simulated waveform, when the DC offset was 0.01 V and *k* = 0.98, the attenuation of the DC offset generated was zero only after two cycles. It was proven that adding the attenuation factor to the transfer function had a certain inhibitory effect on the DC offset.

## 5. Circuit Design

### 5.1. Transmitter

**Figure 10:**
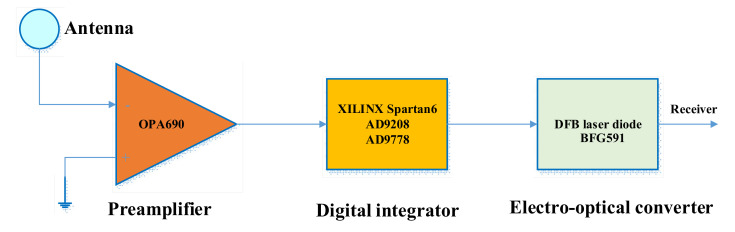
The principle of the transmitter.

The transmitter mainly included three parts: a broadband preamplifier, a digital integrator, and an electro-optical converter, as shown in Figure 10. The preamplifier was a Texas Instruments OPA690; its gain-bandwidth product reaches 400 MHz, and the slew rate can reach 1800 V/μs. It is well suited as an input driver for the ADC. Controlling its enabled terminal can effectively reduce the DC drift of the op-amp. The Spartan-6 is a low-cost, low-power FPGA from Xilinx Products (San Jose, CA, United States). The ADC is an Analog Devices AD9208, which is a 14-bit 3 GSPS analog-to-digital converter. The DAC is an AD9778, which has an update rate of 1 GSPS and 14-bit resolution. The electro-optical conversion adopts a BFG591 broadband triode with a characteristic frequency reaching 7 GHz. We adopted a common emitter connection. The tube was an LSDLD131 DFB (distributed feedback) laser diode, and the wavelength was 1310 nm.

### 5.2. Digital Integration and Frequency Equalization

**Figure 11:**
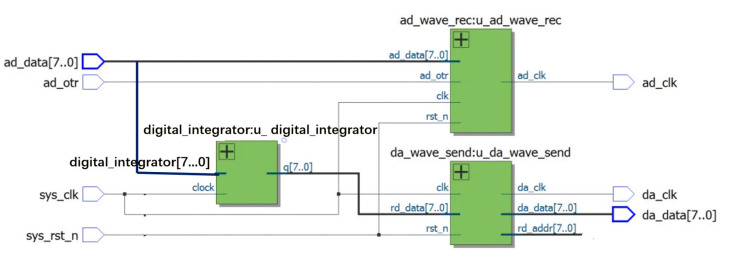
The schematic of the top-level module.

A schematic diagram of the FPGA top-level module is shown in Figure 11. The top-level module instantiated the following three modules: a DA data sending module (da_wave_send), a digital integration module (digital_integrator), and an AD data receiving module (ad_wave_rec).

**Figure 12:**
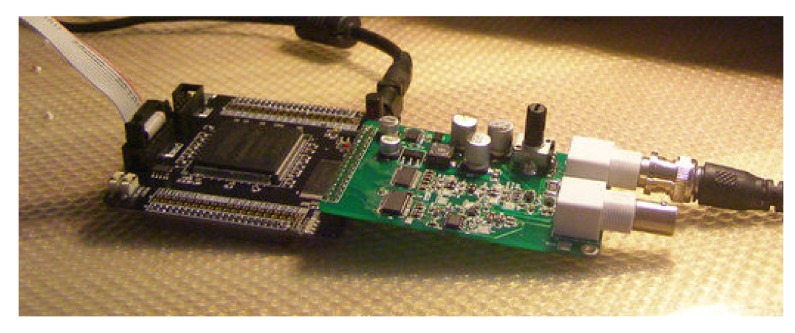
The photo of the digital integrator.

Due to the coupling capacitance, stray capacitance, and stray inductance in the circuit, the low-frequency characteristics of the system were not ideal; in addition, there were still some errors in the high-frequency part of the digital integrator. To ensure that the signal was not distorted as much as possible, we adopted a frequency equalization method to weaken their influence. We used an FIR digital filter because it has the advantages of stable amplitude-frequency characteristics and a strong adaptive ability to avoid the defects of analog filters in practical applications. The basic unit used to implement the FIR filter algorithm includes a storage unit, multiplication unit, an adder, and delay unit. For different compensation filtering effects in different frequency bands to be achieved, a design method of frequency sampling needs to be adopted to design the filter [18,19]. The filter order was 1024, and the coefficients of the filter can be easily designed using FDATOOL (Natick, MA, USA) in MATLAB. After determining the basic implementation structure of the FIR filter, we determined the MAC (multiply accumulation) function on the Xilinx FPGA. The photo of the digital integrator is shown in Figure 12.

### 5.3. Receiver

The photodiode type was an InGaAs PIN; the tube was set at a wavelength of 1310 nm, the conversion coefficient was approximately 1 A/W, and the actual incident light power was 500 μW, and thus the resulting photocurrent was approximately *I*_in_ = 500 μA. The desired output was 4 V, and thus it was necessary to amplify this current by 8 × 10^5^ Ω (transresistance coefficient). It is appropriate to use two-stage amplification, including first-stage transimpedance amplification and second-stage inverting amplification. The magnification of the second stage was set to 80, and then the magnification of the first stage was 10^4^ Ω, that is, the feedback resistance was 10 kΩ. The op-amp was an OPA659, and its gain-bandwidth product can reach 350 MHz; the input noise was as low as $8.9\;\mathrm{nV}/\sqrt{Hz}$, which is suitable for photoelectric amplification. The diode’s dark current *I*_D_, terminal capacitance *C*_t_, and parallel resistance *R*_sh_ directly affect the selection and the size of the noise [20,21].

According to

(6) $$f_{p}=\frac{\left(R_{f}+R_{\mathrm{sh}}\right)}{2\pi R_{f}R_{\mathrm{sh}}(C_{t}+C_{i}+C_{f})}$$

*C_f_* is the phase compensation capacitor; because *R*_sh_ is much larger than *R*_f_, Equation (6) is simplified as

(7) $$f_{p}=\frac{1}{2\pi R_{f}(C_{t}+C_{i}+C_{f})}$$

**Figure 13:**
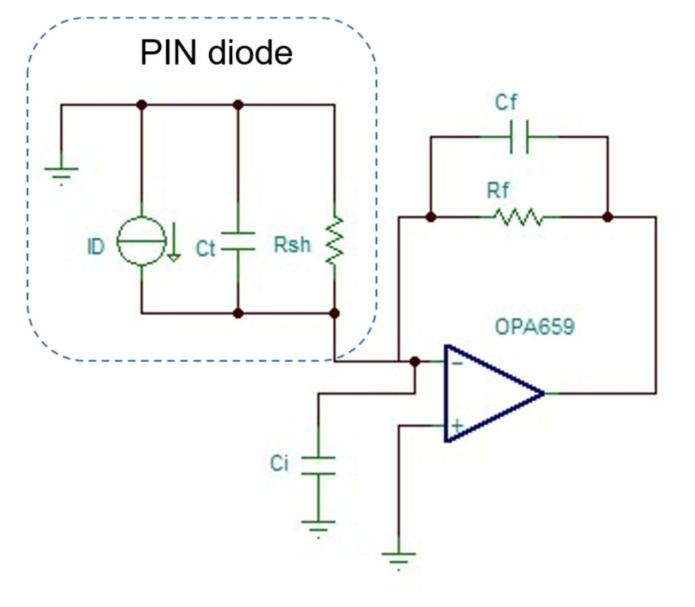
The model of the transimpedance amplifier.

According to Equation (7), the value of the compensation capacitor can be calculated. The equivalent circuit is shown in Figure 13.

**Figure 14:**
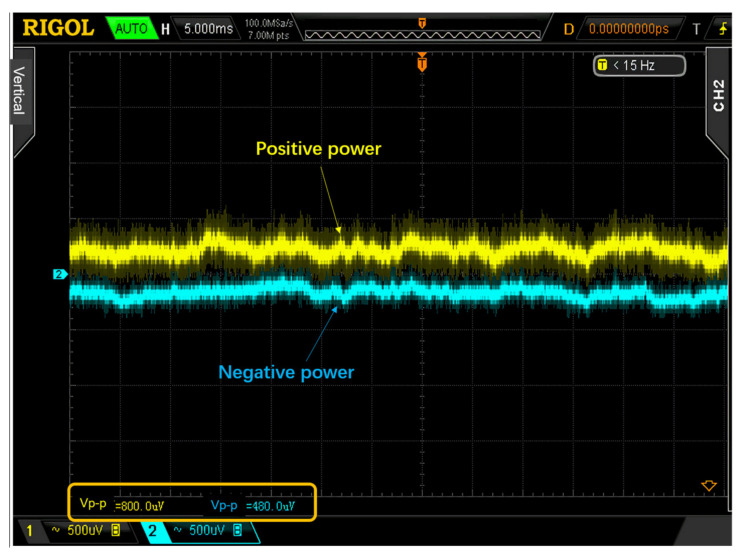
The noise in the power supply.

In terms of power supply, the transmitter was powered by a high-energy density lithium battery to ensure the signal-to-noise ratio of the transmitter. The photodiode is very sensitive to power noise, and therefore the receiver power supply adopted a linear regulator + low noise LDO + CLC filtering. The linear regulator was LM1086CT + LM333T, and the LDO was LT3042 + LT3093, which made the output noise of the entire power supply less than 1 mV, as shown in Figure 14 [22].

**Figure 15:**
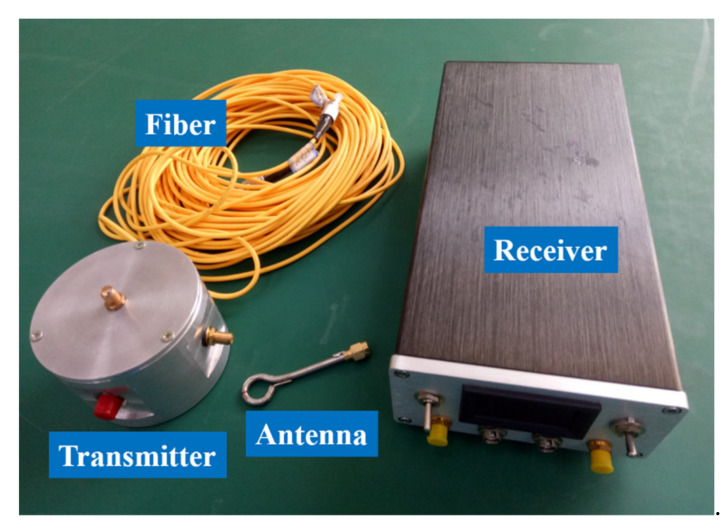
The actual sensor.

The entire sensor is shown in Figure 15. The transmitter adopted a cylindrical design to reduce the disturbance to the surrounding electromagnetic field. Its dimensions were 60 × 60 × 5 mm (diameter × height × thickness).

## 6. Calibration of the Sensor

The IEEE Std 1309-2013 provides three calibration methods: the transfer standard method, standard field method, and reference sensor method [23]. The standard field method mainly uses calculable field strength for calibration. The sensor to be calibrated is placed in a reference field. The reference field is within the frequency band and dynamic range involved in the calibration work. The measured input parameters are calculated and combined to obtain the calibration. This approach replaces the real value with an accurate theoretical value. This article adopted this calibration method.

**Figure 16:**
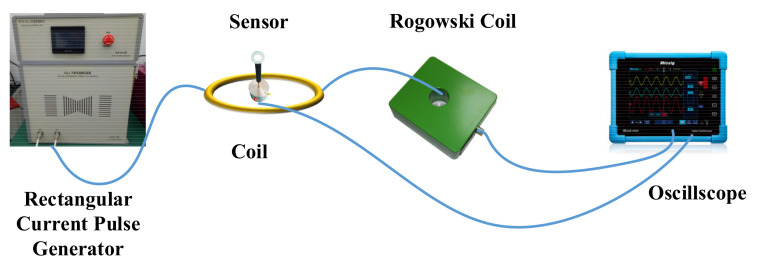
The calibration of magnetic field strength.

The calibration principle is shown in Figure 16. The sensor was placed in the middle of a single-turn coil, and the current generator generated a square current of 0.1–10 kA. A Rogowski Coil (self made) was used for measuring current. We took the theoretical value (*H* = *I*/2*r*, *H*: magnetic field strength, *I*: current, *r*: coil radius) calculation as the true value used for calibration.

**Figure 17:**
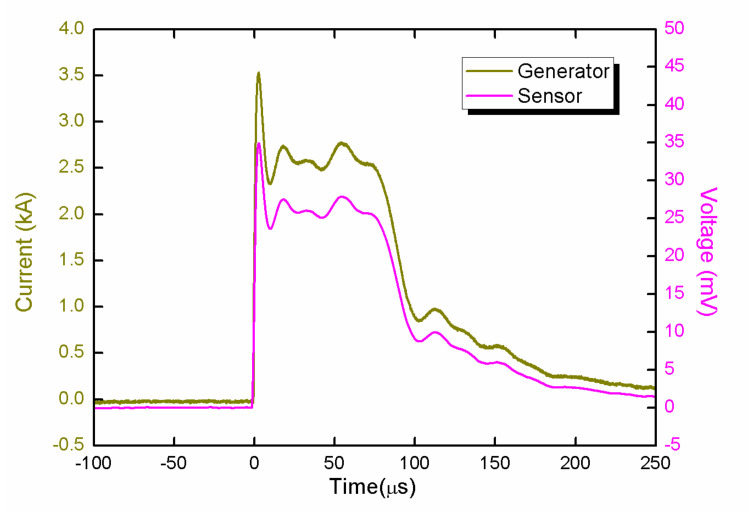
The calibrated waveforms.

As shown in Figure 17, under a 50 μs square current, the magnetic field signal was in good agreement with the current waveform. The magnetic field signal changed slowly in the flat top, but the rise time deviation was within 1%.

### 6.1. Dynamic Range and Linearity

**Figure 18:**
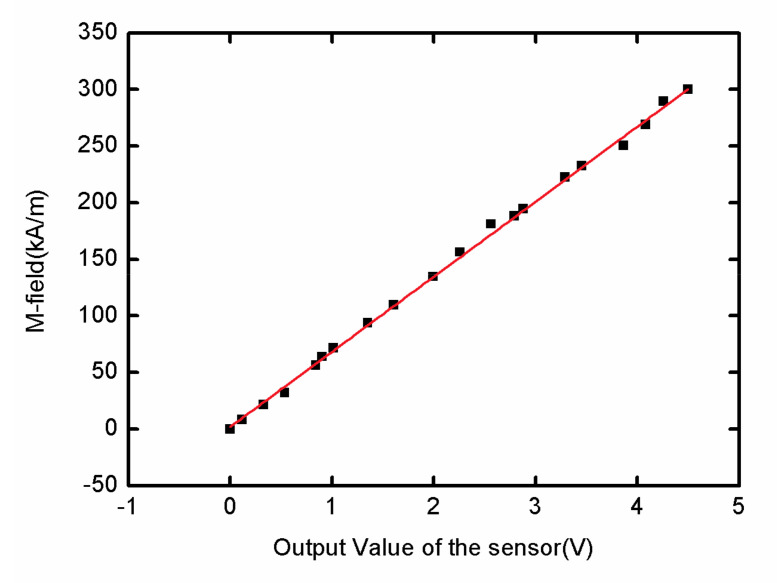
The dynamic range of measurement.

The sensor had a wide dynamic range, from 1 kA/m to 300 kA/m, at approximately 50 dB. Figure 18 shows that the dynamic range of the sensor was improved, especially when the output voltage was in the middle value (1.0–3.5 V), and the linear correlation coefficient was 0.96. The average sensitivity was 68.2 A·m^−1^/mV with linear fitting.

### 6.2. Bandwidth

**Figure 19:**
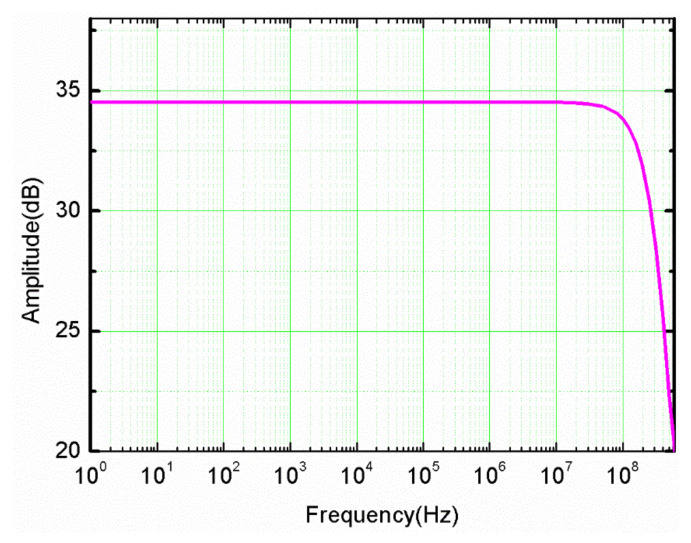
The bandwidth of the sensor.

A Keysight E5061B (Santa Rosa, CA, United States) network analyzer was used to measure the frequency characteristics of the system. Figure 19 shows the measurement result of the bandwidth. The bandwidth reached the expected 100 MHz, and the actual cutoff frequency was 200 MHz. Due to the frequency compensation, the amplitude-frequency response curve was very flat.

### 6.3. Uncertainty Analysis

The measurement uncertainty characterizes the evaluation of the range of the measured true value. The uncertainty of the sensor measurement mainly comes from two aspects: one is the theoretical calculation of the field source, due to the uncertainty caused by the inaccurate measurement of the coil, the inhomogeneity of the field, and the ambient temperature; the other is derived from the test environment, changes in the azimuth angle of the antenna, field distortion caused by the placement of the sensor, measurement error of digital oscilloscope, etc. [24]. Finally, it is necessary to synthesize the above measurement uncertainty components to obtain the synthetic standard uncertainty and extended uncertainty at different frequency points. The uncertainty results calculated at 10 MHz are shown in Table 1.

From the uncertainty estimate, we could see that five standard uncertainties had a greater contribution to the composite uncertainty, four were uniformly distributed, and one was normally distributed. Therefore, the composite standard uncertainty should be close to the uniform distribution, and the corresponding confidence probability is 95%; if the inclusion factor k = 1.65, the expanded uncertainty is

(8) $$U_{c}=ku_{c}=\;2.69$$

## 7. Sensor Application

**Figure 20:**
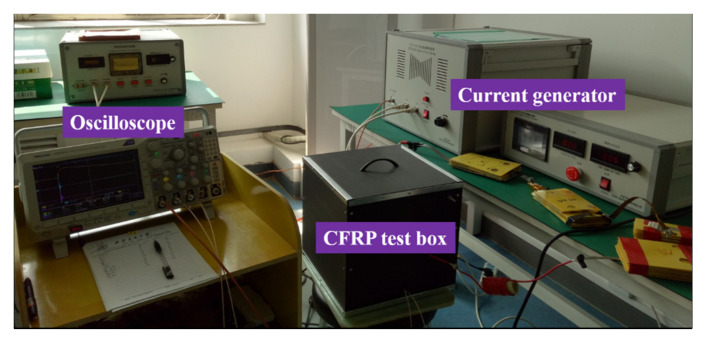
Electromagnetic environment test site (oscilloscope model: Tektronix DPO3014, current sensor model: Pearson 5664).

**Figure 21:**
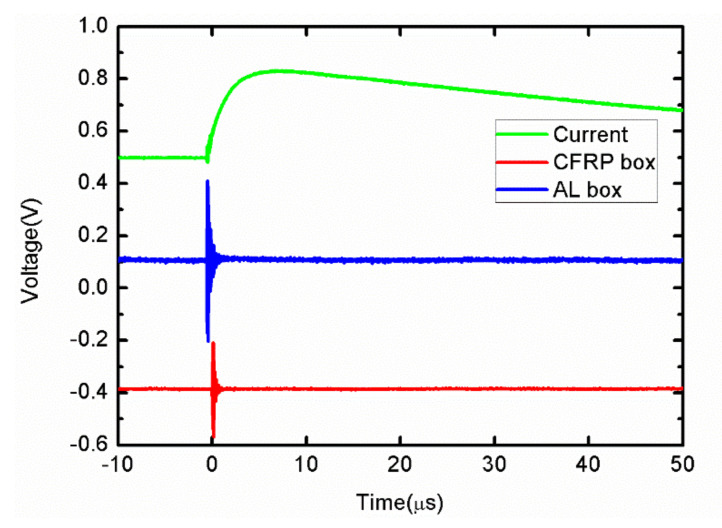
The test waveforms of the electromagnetic environment.

To study the electromagnetic SE of the CFRP, we used an A-type wave current (peak time of 6.4 μs, half peak time of 69 μs, and current peak value of 100 A) for lightning electromagnetic environment testing [25]. As shown in Figure 20, the current was injected into a cubic box made of CFRP and AL alloy. The parameter of the CFRP was four layers, and the laying direction was 45°/0°/–45°/90°. The AL alloy model was 7075, the side length of the box was 300 mm, the thicknesses were both 1 mm, and the path was face-to-face penetration; the electromagnetic field signal of the inner center point was measured. It can be seen in Figure 21 that the two magnetic field signal waveforms were the same as the current waveforms, but the magnetic field strength in the CFRP box was slightly smaller than that of the AL box, and there was a negative spike at the beginning. This was because the CFRP conductivity was three orders of magnitude lower than that of AL, and its SE was lower than that of AL. The electromagnetic wave reflection in the box was weaker. Therefore, the magnetic field strength in the CFRP box was less than that in the AL box as a whole.

## 8. Conclusions

In this paper, a new type of magnetic field sensor was developed, which included a transmitter and a receiver. In the transmitter, the B-DOT antenna was used, and the signal processing used the improved Al-Alaoui digital integration algorithm and the FIR filter frequency equalization method, which not only effectively resisted the DC drift but also made the amplitude-frequency characteristics relatively flat. In the receiver, the photoelectric receiving circuit was carefully designed, and an ultralow noise power supply was used to make the noise floor of the device less than 1 mV. Compared with other magnetic field sensors, due to the optimization of the digital integrator and low-noise design, it had higher measurement accuracy. In addition, the frequency equalization made the frequency band wider; thereby, the measurement uncertainty was reduced.

After experimental testing, the magnetic field waveform and the impulse current waveform were found to be in good agreement. The dynamic range of the sensor was 50 dB (1–300 kA/m), the linear correlation coefficient was 0.96 (68–250 kA/m), the total relative standard uncertainty was 1.63, and the bandwidth was greater than 100 MHz. This approach can be used for lightning electromagnetic environment measurements, electromagnetic radiation measurements in substations, and other occasions where the transient magnetic field needs to be measured. Because the antenna adopts the SMA (Sub-Miniature-A) interface, it is convenient to replace antennas of different sizes or even electric field antennas to indirectly measure the current or electric field.

## Figures and Tables

**Table 1:** Uncertainty sub-table at 10 MHz.

Uncertainty Component	Maximum Deviation (%)	Distribution	Inclusion Factor (k)	Relative Standard Uncertainty (%)
Uncertainty of impulse current	0.2	uniform	$\sqrt{3}$	0.115
Uniformity of field	0.4	uniform	$\sqrt{3}$	0.231
Linearity of the sensor	0.8	uniform	$\sqrt{3}$	0.462
Disturbance of the sensor to the field	1.28	uniform	$\sqrt{3}$	0.739
Measurement error of the coil radius	0.5	normal	1	0.500
Positioning error	2	uniform	$\sqrt{3}$	1.155
Measurement error of Rogowski coil	0.24	normal	1	0.240
Repeatability	0.37	normal	1	0.370
Measurement error of oscilloscope (channel 1)	0.28	uniform	$\sqrt{3}$	0.162
Measurement error of oscilloscope (channel 2)	0.28	uniform	$\sqrt{3}$	0.162
Total relative standard uncertainty *u*_c_				1.63

## Data Availability

Not applicable.

## References

[1] Zhang G., Li W., Qi L., Liu J., Song Z., Wang J. (2018). Design of wideband GHz electric field sensor integrated with optical fiber transmission link for electromagnetic pulse signal measurement. Sensors.

[2] Li Q., Xie Y.-Z., Li K.-J., Kong X. (2019). A portable electric field detector with precise time base for transient electromagnetic radiation source location. IEEE Trans. Instrum. Meas..

[3] Ma M.T., Kanda M., Crawford M.L., Larsen E.B. (1985). A review of electromagnetic compatibility/interference measurement methodologies. Proc. IEEE.

[4] Baum C.E., Farr E.G. (1993). Impulse Radiating Antennas.

[5] Kanda M. (1984). An electromagnetic near-field sensor for simultaneous electric and magnetic-field measurements. IEEE Trans. Electromagn. Compat..

[6] Bose S., Kaur M., Barada K.K., Ghosh J., Chattopadhyay P.K., Pal R. (2019). Understanding the working of a B-dot probe. Eur. J. Phys..

[7] Chao Y., Cui M., Rongmei C., Xin L. Development of high power transient electromagnetic field sensors. Proceedings of the Asia Pacific Symposium on Electromagnetic Compatibility (APEMC).

[8] Kong X., Xie Y.-Z., Li Q., Hu Y.-H. (2020). A multigap loop antenna and norm detector-based nano-second-level transient magnetic-field sensor. IEEE Trans. Instrum. Meas..

[9] Jin Y., Zhang W., Lou J., Wang J., Zhang W. Research of accurate digital integrator for Rogowski coil current transformer. Proceedings of the 2019 IEEE 2nd International Conference on Electronics and Communication Engineering (ICECE).

[10] Han X., Xie J., Luo J., Wu Y., Xu Y. Development of a point coil magnetic field measurement system for pulsed high magnetic fields. Proceedings of the 11th International Conference on Electrical Machines and Systems (ICEMS 2008).

[11] Liu W., Jiang W., Qi H. The design of digital amplitude-frequency equilibrium power amplifier. Proceedings of the International Forum on Information Technology and Applications (IFITA 2010).

[12] Kavehrad M., Lee S., Wu B. (2006). Frequency domain equalization of optical channel distortion in free-space optical wireless communications. Opt. East..

[13] Liu D.-W., Tang Y.-X., Li S.-Q., Da-Wei L., You-Xi T., Shao-Qian L. Performance analysis of TDD OFDM systems with phase and amplitude and phase pre-equalization. Proceedings of the IEEE 60th Vehicular Technology Conference, 2004 (VTC2004-Fall).

[14] Long S., Khalighi M.-A., Wolf M., Ghassemlooy Z., Bourennane S. Performance of carrier-less amplitude and phase modulation with frequency domain equalization for indoor visible light communications. Proceedings of the 2015 4th International Workshop on Optical Wireless Communications (IWOW).

[15] Wei W., Yue S., Xin N., Zhi Z., Jin W., Jing Y. (2020). Development of magnetic field measuring system for fast risetime pulse. High. Volt. Eng..

[16] Kong X., Lei L. (2017). Application of CST electromagnetic simulation technology in antenna experimental teaching. Exp. Technol. Manag..

[17] Bai Y., Wang J., Wei G., Yang Y. (2015). Design and simulation test of an open D-dot voltage sensor. Sensors.

[18] Hu X., Wang J., Wei G., Deng X. (2016). Decomposition of composite electric field in a three-phase D-dot voltage transducer measuring system. Sensors.

[19] Kong X., Xie Y.-Z., Li Q., He S.-Y., Jin Y.-B. (2017). Development of one-dimensional norm detector for nanosecond-level transient electric field measurement. IEEE Trans. Electromagn. Compat..

[20] McKinley A.F. (2017). Theory of impedance loaded loop antennas and nanorings from RF to optical wavelengths. IEEE Trans. Antennas Propag..

[21] Zhang H., Zhou Y., Shi L., Ma R., Huang Z. (2014). Design of electric field sensors for measurement of electromagnetic pulse. Sens. Transducers.

[22] Wang J., Zhao Y., Li W., Zeng X., Tang J., Wang Y., Deng X. (2018). Research on transmission line voltage measurement method of D-dot sensor based on Gaussian integral. Sensors.

[23] Wang K., Duan Y., Shi L., Qiu S. (2020). Laboratory calibration of D-dot sensor based on system identification method. Prime Arch. Sens..

[24] Yan Z., Wang J., Zhang W., Wang Y., Fan J. (2017). A miniature ultrawideband electric field probe based on coax-thru-hole via array for near-field measurement. IEEE Trans. Instrum. Meas..

[25] Holloway C., Sarto M.S., Johansson M. (2005). Analyzing carbon-fiber composite materials with equivalent-layer models. IEEE Trans. Electromagn. Compat..

